# Effectiveness and Impact on Quality of Life of a Hand Rehabilitation Program in Longstanding Systemic Sclerosis: A Prospective, Non-Randomized, Controlled Pilot Study

**DOI:** 10.3390/jcm15187118

**Published:** 2026-09-14

**Authors:** Beatrice Moccaldi, Denisa Pascu, Loriana Esposito, Marco Binda, Anna Cuberli, Andrea Benini, Francesco Carta, Riccardo Verza, Luca Iaccarino, Roberta Ramonda, Elisabetta Zanatta

**Affiliations:** 1 Rheumatology Unit, Department of Medicine—DIMED, University of Padova, 35128 Padova, Italy; beatrice.moccaldi@phd.unipd.it (B.M.); marco.binda@studenti.unipd.it (M.B.); anna.cuberli@studenti.unipd.it (A.C.); andrea.benini@aopd.veneto.it (A.B.); francesco.carta@aopd.veneto.it (F.C.); luca.iaccarino@unipd.it (L.I.); roberta.ramonda@unipd.it (R.R.); 2 Department of Neurosciences, University of Padova, 35121 Padova, Italy; denisa.pascu@studenti.unipd.it; 3 Rehabilitation Center “Fisio Medica Soncin”, 35122 Padova, Italy; lorianaesposito@libero.it; 4 Istituto di Ricovero e Cura a Carattere Scientifico (IRCCS) E. Medea—Ass. La Nostra Famiglia, 31015 Conegliano, Italy; riccardo.verza@unipd.it; 5 Department of Surgery, Oncology and Gastroenterology—DISCOG, University of Padova, 35128 Padova, Italy; 6 Department of Cardio-Thoraco-Vascular Sciences and Public Health, University of Padova, 35128 Padova, Italy

**Keywords:** systemic sclerosis, physical therapy, hand rehabilitation, quality of life, joint contracture

## Abstract

**Background/Objectives:** Reduced hand function significantly contributes to disability in patients with systemic sclerosis (SSc). We aimed to evaluate the effects on hand function and quality of life of a multicomponent hand rehabilitation program in patients with longstanding SSc. **Methods:** This prospective, controlled, interventional pilot study enrolled adults with SSc, disease duration >5 years and ≥1 upper limb joint contracture. The intervention consisted of 10 sessions over 5 weeks, including paraffin wax therapy and individualized hand rehabilitation. The allocation to the intervention or control group was based on the patients’ availability to participate in the program. Hand mobility/function and health-related quality of life were assessed through validated outcome measures at baseline and after 5 weeks. **Results:** Sixteen patients were included (intervention *N* = 10; control *N* = 6), with a median disease duration of 19.5 (15.3–24.3) years. Within the intervention group, significant improvements were observed in right-hand Hand Mobility in Scleroderma (HAMIS) score (*p* = 0.033) and in the Short Form (SF)-36 domains of “Energy/fatigue” (*p* = 0.008), “Emotional well-being” (*p* = 0.009), “Pain” (*p* = 0.020), and “General health” (*p* = 0.050). Compared with controls, significant benefit was observed in the SF-36 “Energy/fatigue” (*p* = 0.002; *d* = 1.97) and “General health” (*p* = 0.011; *d* = 1.50) domains. Between-group differences in hand function measures did not reach statistical significance, although moderate-to-large effect sizes favored rehabilitation across several outcomes. **Conclusions:** In this pilot study, a multicomponent hand rehabilitation program was associated with improvements in quality of life in patients with longstanding SSc, despite limited effects on hand mobility. These preliminary findings suggest a potential benefit of hand rehabilitation even in advanced disease.

## 1. Introduction

Systemic sclerosis (SSc) is a connective tissue disease characterized by widespread fibrosis of the skin and internal organs [1]. In SSc, internal organ involvement, particularly cardiac and pulmonary complications, is the leading cause of disease-related mortality [2]. However, skin thickening and musculoskeletal symptoms—including arthritis/arthralgia, joint contractures, calcinosis and myopathy—significantly contribute to disability and impaired quality of life in SSc patients [3]. In particular, joint contractures are often responsible for reduced mobility and represent irreversible disease damage; therefore, they are included as a specific item in the Systemic Sclerosis Clinical Trials Consortium (SCTC) Damage Index [4,5]. The hands are affected in a substantial proportion of SSc patients, resulting in reduced hand function, which is a major determinant of decreased health-related quality of life [6].

While immunosuppressive treatment is crucial during the early stages of the disease to reduce the inflammatory burden—responsible for musculoskeletal symptoms such as arthritis and synovitis, as well as skin thickening—this approach primarily targets active inflammation to prevent early damage accrual [7]. However, as the disease progresses, non-inflammatory mechanisms become the predominant drivers of ongoing hand function impairment. In the later stages, factors such as periarticular tissue fibrosis, vasculopathy, skin atrophy and osteolysis contribute substantially to functional decline [8,9,10,11]. These changes reflect irreversible structural damage rather than active inflammation, highlighting the need for comprehensive management strategies addressing fibrosis and tissue remodeling.

The role of hand rehabilitation has been increasingly investigated in SSc patients, with the goals of preserving and improving hand function, reducing disability, and enhancing overall quality of life. The latest EULAR guidelines on non-pharmacological management of SSc underline the importance of hand exercises for the improvement of hand function in affected patients [12]; moreover, in 2022, a Dutch task force elaborated a consensus-based set of recommendations on non-pharmacological treatment of hand function loss in SSc, highlighting that patient education should take into account information about regular hand exercise, either independently and under the guidance of a skilled health professional (e.g., hand therapist), with multidisciplinary rehabilitation to be considered in selected patients [13].

Several small studies have explored different hand exercise protocols in SSc patients [14,15,16,17], but most existing research focuses on patients with early systemic sclerosis (i.e., disease onset within 5 years), in whom musculoskeletal alterations may still be reversible with immunosuppressive therapy. In contrast, there is a lack of studies addressing individuals with longstanding disease and advanced, irreversible damage, who represent a population with significant unmet therapeutic needs [18]. Therefore, we aimed to design an intensive hand physical therapy program specifically targeting patients with longstanding SSc, to evaluate the benefit on the improvement of hand function and quality of life in this underrepresented group.

## 2. Materials and Methods

### 2.1. Study Design

We designed a prospective, non-randomized, controlled, interventional study with the aim of evaluating the effect of a multicomponent hand rehabilitation program on hand function and health-related quality of life in patients with longstanding SSc and joint contractures. Outcome measures of hand function and quality of life were assessed at baseline (T0), prior to initiation of the intervention, and after 5 weeks (T1), corresponding to the end of the treatment period in the intervention group, with matched time points for the controls. Since recruitment was feasibility-based due to limited number of eligible patients, no formal sample size calculation was performed and no primary endpoint was prespecified. Accordingly, all analyses were considered exploratory and hypothesis-generating. The study involved a multidisciplinary team, including rheumatologists (who were responsible for recruitment and assessment of outcome measures), a physical medicine specialist (who evaluated patients at enrollment), and physiotherapists (who delivered the rehabilitation program and contributed to the assessment of outcome measures). Outcome assessors were not blinded to group allocation.

The study was conducted in accordance with the Declaration of Helsinki and approved by the local Ethics Committee of the University Hospital of Padova (protocol number 6499/AO/25). All participants provided written informed consent prior to inclusion.

### 2.2. Participants

Participants were recruited from the cohort of SSc patients followed at the Rheumatology Unit of the University Hospital of Padova. Eligible participants were adults who fulfilled the 2013 ACR/EULAR classification criteria for SSc [19], had a disease duration >5 years (from the first non-Raynaud’s sign or symptom) at the time of enrolment, and presented with at least one upper limb joint contracture, defined as any degree of contracture with the inability to reduce the joint to the anatomically neutral position [4]. Patients on immunosuppressive treatment were required to be on a stable dose for at least 3 months upon enrollment.

Exclusion criteria were the presence of active inflammatory arthritis related to SSc, overlap with rheumatoid arthritis, recent fractures involving the upper limbs, previous surgery significantly limiting joint range of motion in the upper limbs, and current participation in other structured hand rehabilitation programs.

All eligible patients were offered participation in the rehabilitation program. Patients who accepted and were able to attend the scheduled rehabilitation sessions constituted the intervention group, whereas patients who were unable to attend the therapy sessions, but agreed to take part in the evaluations, served as the control group. Eligible patients who did not provide informed consent or were unable to participate in either in the intervention or the control group were excluded.

### 2.3. Intervention

The rehabilitation protocol consisted of a multicomponent intervention of paraffin wax therapy and hand physical therapy. Ten treatment sessions were conducted over 5 weeks (twice weekly), with a total duration of approximately 45 min for each treated hand. Rehabilitation was delivered by five physiotherapists with great experience in hand rehabilitation. The first phase of each session consisted of paraffin wax therapy administered for 20 min. Paraffin was applied using the traditional multiple-dip immersion technique to ensure uniform heat distribution over the distal segments of the upper limb. In patients presenting with digital ulcers or trophic skin alterations, the treatment was performed using appropriate protective barriers consisting of sterile gauze and vinyl gloves to prevent direct contact between the paraffin and the lesions while still providing a controlled and homogeneous thermal effect. The remaining 25 min of the session were dedicated to specific hand physical therapy, with both hands being treated during each session. Active, active-assisted, and passive joint mobilization exercises were performed involving the fingers, metacarpophalangeal joints, interphalangeal joints, thumb, and wrist, with particular emphasis on restoring and maintaining joint range of motion. A fixed number of repetitions was not prescribed: joint mobilizations were performed progressively and within each patient’s tolerance. The treatment also included manual therapy and superficial soft tissue mobilization techniques targeting the skin and subcutaneous tissues of the hands and fingers. These consisted of superficial tissue release, gentle kneading, and skin mobilization techniques aimed at improving tissue gliding and enhancing skin mobility relative to the underlying structures. No home exercise was prescribed.

The rehabilitation intervention was individualized according to each patient’s clinical condition and the presence of pain, edema, skin contractures, or joint stiffness. The occurrence of any treatment-related adverse events, such as burns, ulcer deterioration or pain exacerbation during treatment, was recorded.

### 2.4. Outcome Measures

All participants underwent a complete evaluation of outcome measures at baseline (T0) and after 5 weeks (T1). The study focused on three main domains: hand mobility, hand and upper limb function, and overall quality of life.

Hand mobility was assessed using both a performance-based and an objective clinical measure. The Hand Mobility in Scleroderma (HAMIS), specifically developed for SSc, evaluates nine standardized movements involving the fingers, wrist, and forearm, including flexion, extension, and grip-related tasks [20]. Each item is scored from 0 (no impairment) to 3 (severe impairment), with higher scores indicating greater functional limitation. In addition, finger flexion was quantified using the finger-to-palm (FTP) distance, expressed in centimeters, which provides a simple and objective measure of residual range of motion [21].

Hand and upper limb function were evaluated using validated patient-reported outcome measures. The Cochin Hand Function Scale (CHFS), a questionnaire specific for SSc, was used to assess difficulty in performing daily activities requiring manual dexterity. It consists of 18 items scored from 0 (no difficulty) to 5 (impossible to perform), with higher scores reflecting greater disability [22]. Broader upper limb function and related symptoms were assessed using the Disabilities of the Arm, Shoulder and Hand (DASH) questionnaire, a 30-item instrument generating a score from 0 (no disability) to 100 (most severe disability) [23]. General functional ability was further evaluated using the Health Assessment Questionnaire (HAQ), which measures difficulty in performing activities of daily living across multiple domains. Each category includes questions rated on a scale from 0 (no difficulty) to 3 (unable to do), with the highest score in each category used to calculate an overall disability score [22].

Quality of life and general health status were assessed using the Short Form-36 (SF-36), a widely used generic instrument that evaluates both physical and mental health domains, including physical functioning, pain, vitality, emotional well-being, and social functioning; each domain receives a score between 0 and 100%, with higher scores indicating better quality of life [24].

All instruments were used in their validated Italian versions [25,26,27,28,29].

In addition to outcome measures, demographic and disease-related variables were collected at baseline, including age, sex, anthropometric data, disease duration, disease subset [30], ongoing immunosuppressive therapy, and organ involvement. Specific clinical features such as modified Rodnan Skin Score (mRSS), the presence of interstitial lung disease, cardiac and gastrointestinal involvement, history of digital ulcers, skin fibrosis, myositis, arthritis, tendon friction rubs, calcinosis, and the number and distribution of joint contractures were recorded.

### 2.5. Statistical Analysis

Continuous variables were expressed as median and interquartile range (IQR), whereas categorical variables were reported as absolute frequencies and percentages. Given the small sample size and to limit reliance on distribution assumptions, baseline features were compared between the intervention and control groups using the Mann–Whitney U test for continuous variables and Fisher’s exact test for categorical variables. Within-group changes between baseline (T0) and follow-up (T1) were analyzed using Wilcoxon signed-rank test. For the primary between-group analysis, the estimand of interest was the difference in mean change from baseline. Accordingly, individual changes (Δ = T1 − T0) were calculated for each outcome and summarized as mean change with 95% confidence interval (95% CI). Differences in mean changes over the study period between the two groups were assessed using an independent-samples *t*-test. Homogeneity of variance was assessed using Levene’s test and, in case of evidence of unequal variances, Welch’s *t*-test was used. Between-group effect size was estimated using the standardized mean difference (SMD), expressed as Cohen’s d with corresponding 95% CI. The distribution of individual changes was examined graphically using Q-Q plots. Considering the small sample size and the deviations from normality observed for some outcomes, Mann–Whitney U tests were additionally performed as sensitivity analyses to assess the robustness of the primary between-group findings. Given the small sample size and the feasibility-based design, no baseline-adjusted analysis or correction for multiple comparisons was performed. All statistical tests were two-sided, with a *p*-value < 0.05 considered statistically significant. All reported *p*-values are unadjusted; accordingly, all findings should be considered exploratory and hypothesis-generating.

The statistical analysis was performed using Jamovi Statistical Software (The jamovi project (2026), Version 2.3.26.0, Sydney, Australia) and R Statistical Software (R Core Team (2025). R: A Language and Environment for Statistical Computing, Version 4.5.2, R Foundation for Statistical Computing, Vienna, Austria).

## 3. Results

### 3.1. Demographic, Clinical and Serological Features of the Study Population

**Figure 1:**
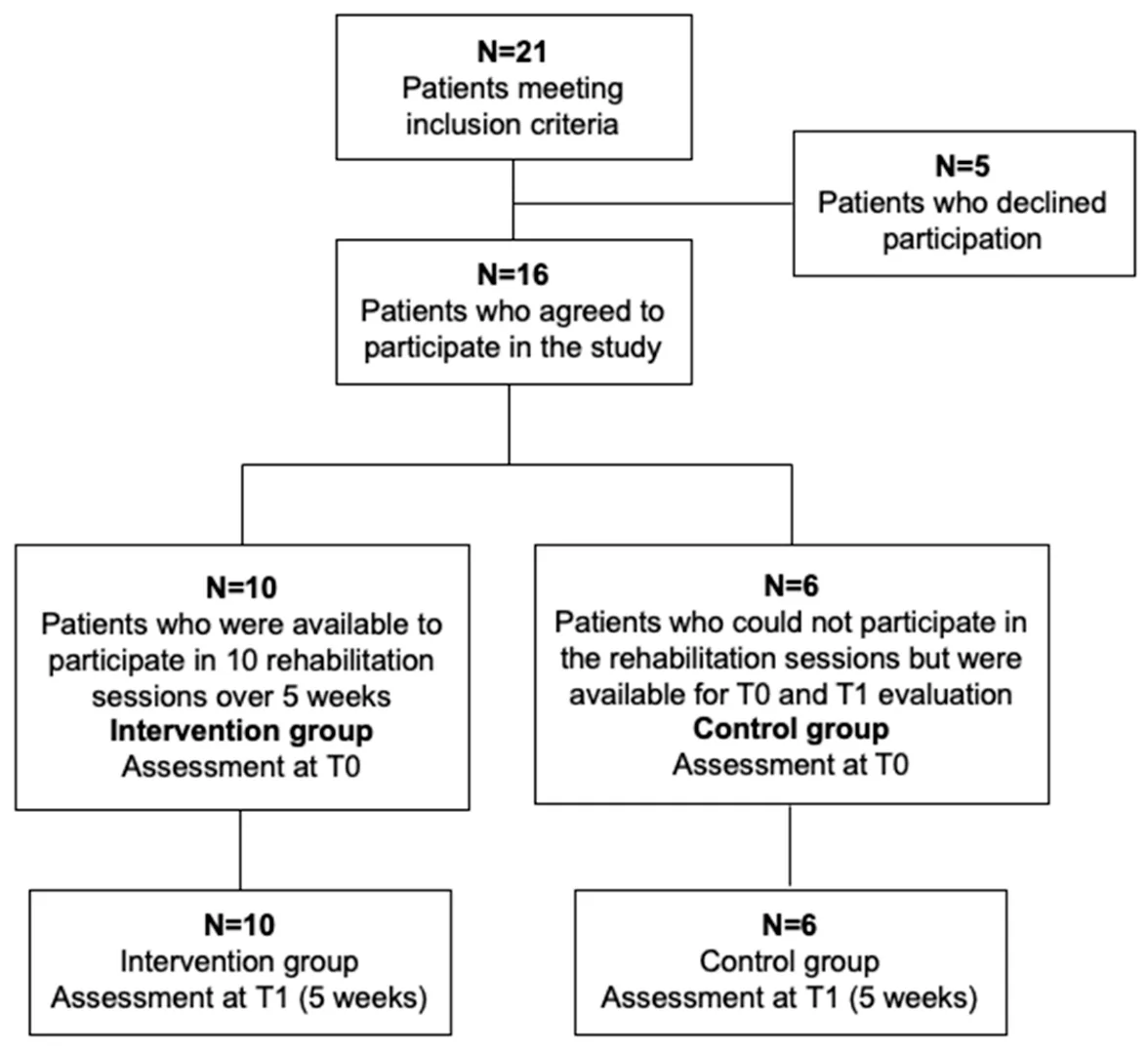
Study design.

Of the 21 patients who met the eligibility criteria, 5 declined participation in the study and were therefore excluded. Of the 16 patients who provided informed consent, 10 accepted and were able to participate in the rehabilitation sessions, thus constituting the intervention group; six patients were unable to attend the rehabilitation sessions but agreed to undergo T0 and T1 assessments, thus constituting the control group. All patients in the intervention group were able to attend the 10 rehabilitation sessions and all patients in the control group were available for the evaluation at 5 weeks, as shown in Figure 1. The majority of participants were female (94%), with a median age of 55.5 (53–61) years and a median disease duration of 19.5 (15.3–24.3) years. Ten patients (62%) had diffuse cutaneous SSc (dcSSc), and 12 (75%) were positive for anti-topoisomerase I antibodies (ATA). Internal organ involvement was common, with interstitial lung disease in 13 patients (81%), primary heart involvement in 3 (19%), pulmonary arterial hypertension in 1 (6%), and gastrointestinal involvement in 10 (62%).

Musculoskeletal manifestations were also frequent. At study entry, 10 patients (62%) reported arthralgia, whereas one patient each (6%) had clinically evident myositis and tendon friction rubs (TFR). All participants presented with at least one upper limb joint contracture; most contractures involved the small joints of the hands, while three patients also had elbow contractures. The median number of joint contractures was 8 (8–10). No significant differences were observed between the intervention and control groups regarding demographic, clinical, serological characteristics, or concomitant therapies (Table 1).

### 3.2. Within-Group Changes

Baseline values of all functional, disability, and health-related quality of life outcome measures are reported in Table 2. Most comparisons did not reach statistical significance; however, the SF-36 “Social Functioning” domain differed significantly between groups at baseline (*p* = 0.047) and some other numerical differences were observed across baseline outcome measures, such as the HAMIS and other SF-36 domains. Changes in outcome measures from baseline (T0) to follow-up (T1) are summarized in Table 3. Within the intervention group, a significant reduction in the HAMIS score was observed for the right hand [12.5 (IQR 9.25–15.00) vs. 10.0 (IQR 8.25–11.00), *p* = 0.033], whereas no significant change was detected for the left hand. Finger-to-palm distance, CHFS, DASH, and HAQ scores remained stable over the study period. Regarding health-related quality of life, patients enrolled in the rehabilitation program experienced significant improvements in several SF-36 domains. Specifically, “Energy/fatigue” increased from a median of 45 to 67.5% (*p* = 0.008), “Emotional well-being” from 70 to 86% (*p* = 0.009), “Pain” from 45 to 60% (*p* = 0.020), and “General health” from 27.5 to 32.5% (*p* = 0.050). No significant improvement was reached in the “Physical functioning”, “Role limitations due to physical health”, “Role limitations due to emotional problems” and “Social functioning” domains. In contrast, no significant longitudinal changes were observed in the control group for any of the evaluated outcome measures. Nevertheless, a trend towards worsening disability was noted, as reflected by increases in CHFS and HAQ scores, although these differences did not reach statistical significance.

Notably, no treatment-related adverse events, especially in relation to paraffin wax therapy, were observed during the study.

### 3.3. Between-Group Comparisons

**Figure 2:**
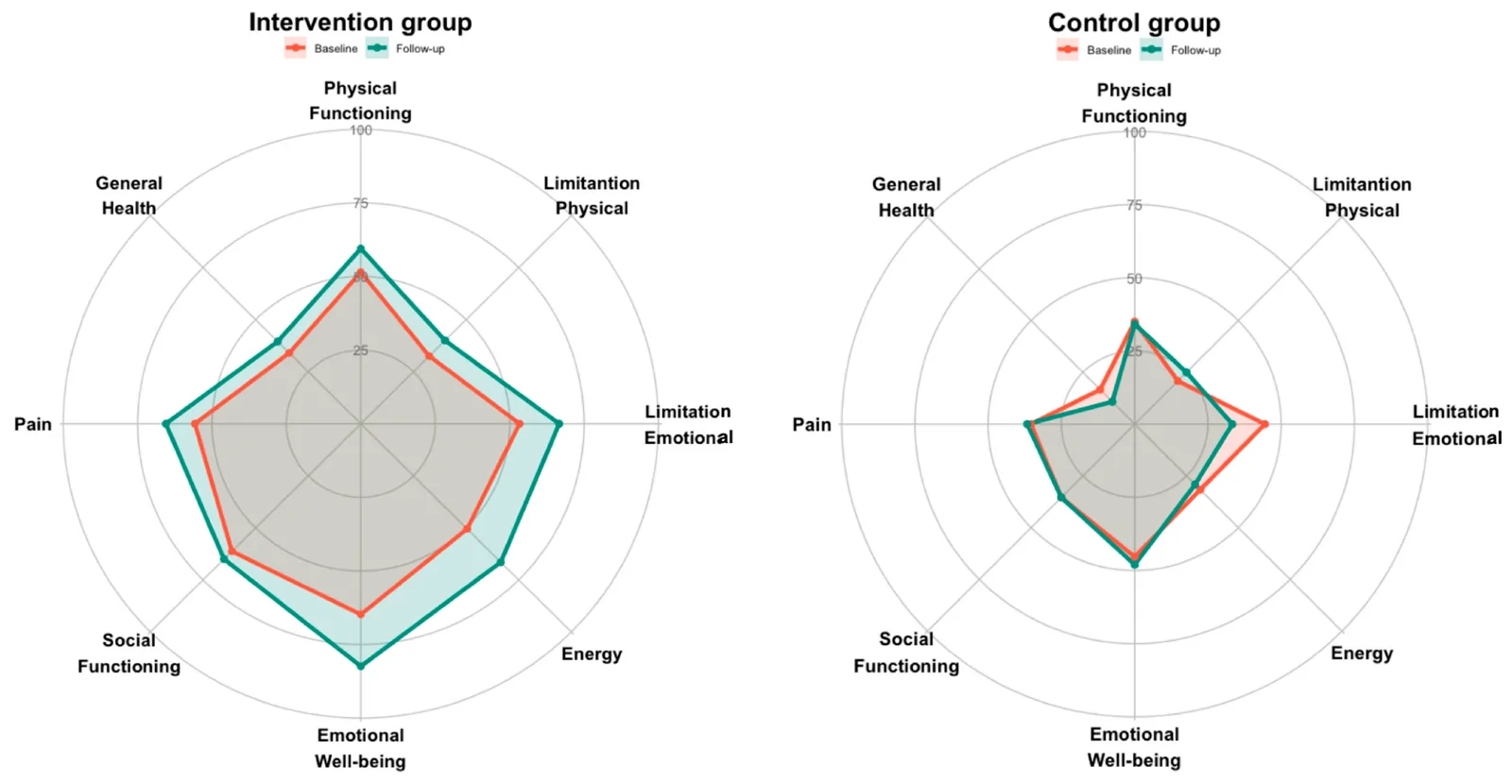
Radar plot of changes in SF-36 domains in the intervention vs. control group.

Between-group comparisons of changes from baseline (T0) to follow-up (T1) are presented in Table 4. Patients allocated to the rehabilitation program showed significantly greater improvements than controls in “Energy/fatigue” domain [mean change +16% (95% CI 7.95 to 24.05) vs. −2.5% (95% CI −6.89; 1.89), *p* = 0.002)] and in the “General health” domain [mean change +5.5% (95% CI 0.32; 10.68) vs. −5.83% (95% CI −14.24; 2.57), *p* = 0.011] of the SF-36, corresponding to large standardized effect sizes [Cohen’s d = 1.97 (95% CI 0.38; 3.50), and 1.50 (95% CI 0.11; 2.83), respectively]. The remaining comparisons did not reach statistical significance, though several outcomes showed moderate-to-large effect sizes favoring the intervention. These included right-hand HAMIS (d = −1.02), CHFS (d = −1.08), and the SF-36 domains of “Role Limitations due to Emotional Problems” (d = 0.99), “Emotional Well-being” (d = 0.90), and “Pain” (d = 0.94). By contrast, changes in finger-to-palm distance, DASH, HAQ, left-hand HAMIS and the remaining SF-36 domains showed small-to-moderate effect sizes. The sensitivity analysis revealed the same pattern of statistical significance as the primary analysis, with significant between-group differences observed only for the SF-36 “Energy/fatigue” and “General Health” domains (*p* = 0.002 and *p* = 0.021, respectively), as shown in Supplementary Table S1. The changes in the SF-36 domains over the study period in the two groups are illustrated in Figure 2.

## 4. Discussion

In this single-center prospective interventional pilot study, we aimed to evaluate the efficacy on hand function and the impact on quality of life of a 5-week multicomponent rehabilitation program targeting the hands and the upper limbs in patients with longstanding SSc. Musculoskeletal involvement, particularly affecting the hands, is a major contributor to disease-related disability and impaired quality of life in SSc patients [6]. Despite being recognized as a potentially valuable component in the multidisciplinary management of SSc [11,12,31], hand rehabilitation is not yet routinely recommended in clinical practice, mainly due to the limited available evidence. Our study provides further support for its implementation, and highlights the need for future research aimed at optimizing rehabilitation protocols and identifying the patients most likely to benefit.

Overall, our findings suggest that, in this subgroup of patients with advanced disease and established musculoskeletal damage, this approach may positively influence patients’ perception of health status and quality of life, despite having a relatively limited impact on objective measurements of hand mobility.

The most relevant findings of our study are the between-group differences observed in several domains of the SF-36 questionnaire, specifically “Energy/fatigue” and “General health”, with large effect sizes favoring the intervention group, although with wide confidence intervals, reflecting the limited precision of these estimates. Improvements were also observed in other SF-36 domains (“Role Limitations due to Emotional Problems”, “Emotional Well-being” and “Pain”), although with smaller effect sizes. It is worth noting that mean improvements in the intervention group exceeded the 5-point threshold for clinically meaningful change, which has been reported in other studies on SSc, in seven out of the eight SF-36 domains [32,33]. These results suggest that the benefits of rehabilitation may extend beyond the mechanical improvement of joint mobility, positively influencing patients’ perception of their health, pain burden and overall well-being. Such findings are clinically relevant, considering that, in our cohort, baseline SF-36 scores were consistently low across nearly all domains, emphasizing the substantial burden imposed by longstanding disease on patients’ physical and psychological well-being [34]. Importantly, the majority of our patients received immunosuppressive therapy during the observation, suggesting that optimization of pharmacological treatment alone does not necessarily translate into significant improvements in health-related quality of life; therefore, the additional benefit observed following rehabilitation supports its role in addition to medical therapy. In this context, even modest improvements in patient-reported outcomes may represent meaningful clinical benefits.

By contrast, between-group differences in objective measures of hand function and mobility were less pronounced. Nevertheless, several outcomes—particularly right-hand HAMIS and CHFS—showed moderate-to-large standardized effect sizes favoring the intervention, suggesting that the study may have been underpowered to detect significant differences in these outcomes. The finding that improvement in HAMIS scores was observed only for the right hand should be interpreted with caution, as it may represent a chance finding related to the small sample size. Moreover, the trend toward worsening CHFS and HAQ scores observed in the control group may suggest that rehabilitation could help to preserve hand function, potentially mitigating the progressive functional decline occurring over the natural course of SSc [35]. However, this observation remains speculative as the difference did not reach statistical significance, and this finding needs to be confirmed in future studies.

Our findings should also be interpreted in light of the unique characteristics of the study population. Unlike most previously published studies, which mainly included patients with early SSc (disease duration < 5 years) [14,16], our cohort consisted exclusively of patients with longstanding disease, with a mean disease duration over 20 years, and established irreversible musculoskeletal damage. In our cohort, the benefits on objective hand function and mobility appeared more limited than those obtained in early SSc cohorts [14]. It bears noting that, at this stage of the disease, significant improvements in joint mobility may not be expected after a relatively short rehabilitation program; thus, patients with longstanding structural damage may require longer rehabilitation to achieve measurable functional gain [36]. Instead, physical therapy may primarily improve patients’ adaptation to chronic disability, optimize residual function and reduce the perceived burden of disease.

Taken together, our findings support the role of rehabilitation even in this challenging subgroup of patients, while suggesting that its early initiation—before irreversible joint contractures occur—may maximize its impact on hand function. These observations further strengthen the rationale for the EULAR recommendations [12], which advocate rehabilitation as part of the multidisciplinary care of SSc, extending the available evidence to patients with longstanding disease, a population that has been underrepresented in previous studies.

Our study has several strengths. To our knowledge, this is the first study to evaluate a structured hand rehabilitation program specifically in patients with longstanding SSc. Moreover, the study design with the presence of a control group allows determination of the effect size of the intervention. Furthermore, the rehabilitation protocol was delivered by experienced physiotherapists along with a multidisciplinary team and the assessment included both objective clinical assessments and validated patient-reported outcome measures, providing a comprehensive evaluation of treatment effects.

We would be remiss not to mention some limitations of our study. First, the small sample size may have reduced the statistical power of our observations and precluded adjustment for potential confounders, rendering our findings exploratory and hypothesis-generating. This limitation reflects the difficulty of recruiting a highly selected subgroup of patients with a rare disease, namely SSc patients with longstanding disease and joint contractures. Second, group allocation was not randomized but depended on the patients’ ability to participate in the rehabilitation sessions, potentially introducing a selection bias; moreover, especially given the small sample size, potential baseline imbalances between the groups, including a significant difference in SF-36 Social Functioning, cannot be excluded as a source of bias. Third, neither participants nor outcome assessors were blinded to the treatment allocation: although blinding was not feasible due to the nature of the intervention, this may have introduced expectancy and response bias, which may have influenced patient-reported outcome measures, such as the SF-36. Finally, the treatment period was relatively short and post-treatment follow-up was not available, thus precluding the evaluation of persistence of treatment effects over time.

## 5. Conclusions

Our pilot study suggests that a multicomponent hand rehabilitation program may be associated with improved health-related quality of life in patients with longstanding SSc, despite showing limited changes in objective measures of hand mobility. These preliminary findings suggest a potential benefit of hand rehabilitation even in advanced disease, thus supporting the integration of structured rehabilitation into the multidisciplinary management of this subgroup of SSc patients. At the same time, the limited functional improvement observed in our cohort, compared with previous studies in early SSc, may indicate that rehabilitation should be introduced earlier in the disease course, before irreversible structural damage develops, when its impact on hand function is likely to be greater. Larger studies are warranted to confirm these preliminary observations, identify the patients most likely to benefit from rehabilitation, and define the optimal timing, intensity, and duration of treatment. Moreover, studies with longer follow-up are necessary to determine whether the observed improvements are sustained over time, or whether longer or maintenance rehabilitation programs may provide additional benefits, particularly in patients with longstanding disease.

## Figures and Tables

**Table 1:** Clinical and demographic features of the study population.

	All Patients (*N* = 16)	Intervention Group (*N* = 10)	Control Group (*N* = 6)	*p*
Age, y (IQR)	55.5 (53–61)	56 (52.5–70)	55 (53.3–58.3)	0.744
Sex F, *n* (%)	15 (94)	10 (100)	5 (83)	>0.99
Disease duration, y (IQR)	19.5 (15.3–24.3)	18.5 (11.5–22)	22 (20–27.8)	0.115
dcSSc, *n* (%)	10 (62)	6 (60)	4 (67)	>0.99
ATA, *n* (%)	12 (75)	9 (90)	3 (50)	0.118
ACA, *n* (%)	1 (6)	1 (10)	0	>0.99
ARA, *n* (%)	1 (6)	0	1 (17)	0.375
mRSS (IQR)	12 (9–15.3)	12 (11–13.8)	12.5 (7.5–17.5)	0.870
ILD, *n* (%)	13 (81)	9 (90)	4 (67)	0.518
Cardiac, *n* (%)	3 (19)	2 (20)	1 (17)	>0.999
PAH, *n* (%)	1 (6)	1 (10)	0	>0.999
GI involvement, *n* (%)	10 (62)	7 (70)	3 (50)	0.607
DUs ever, *n* (%)	9 (56)	5 (50)	4 (67)	0.633
Amputation, *n* (%)	1 (6)	0	1 (17)	0.375
Calcinosis, *n* (%)	8 (50)	4 (40)	4 (67)	0.608
Arthralgia,				
at T0, *n* (%)	10 (62)	7 (70)	3 (50)	0.607
ever, *n* (%)	11 (69)	7 (70)	4 (67)	>0.99
Myositis,				
at T0, *n* (%)	1 (6)	1 (10)	0	>0.99
ever, *n* (%)	1 (6)	1 (10)	0	>0.99
TFR,				
at T0, *n* (%)	1 (6)	1 (10)	0	>0.99
ever, *n* (%)	2 (12)	2 (20)	0	0.500
Joint contractures at T0, n (IQR)	8 (8–10)	8 (6.5–10)	8 (8–9.5)	0.733
Ongoing IS at T0				
Glucocorticoids, *n* (%)	8 (50)	5 (50)	3 (50)	>0.99
MTX, *n* (%)	1 (6)	1 (10)	0	>0.99
MMF, *n* (%)	9 (56)	4 (40)	5 (83)	0.145
TCZ, *n* (%)	2 (12)	1 (10)	1 (17)	>0.99
RTX, *n* (%)	5 (31)	3 (30)	2 (33)	>0.99

ACA, anticentromere antibodies; ARA, anti-RNA polymerase III antibodies; ATA, antitopoisomerase I antibodies; dcSSc, diffuse cutaneous Systemic Sclerosis; DUs, digital ulcers; F, female; GI, gastrointestinal; ILD, interstitial lung disease; IQR, interquartile range; IS, immunosuppressants; MMF, mycophenolate mofetil; mRSS, modified Rodnan Skin Score; MTX, methotrexate; PAH, pulmonary arterial hypertension; RTX, rituximab; TCZ, tocilizumab; TFR, tendon friction rubs.

**Table 2:** Baseline (T0) assessment of outcome measures in the intervention and control groups.

	All Patients (*N* = 16)	Intervention Group (*N* = 10)	Control Group (*N* = 6)	*p*
HAMIS (IQR)				
Right	13.5 (10–17,5)	12.5 (9.25–15)	17 (12–22)	0.229
Left	12.5 (8.75–18.75)	10.5 (7.25–13)	17.5 (12.5–21.75)	0.073
FTP distance, cm (IQR)				
Right	4.75 (3.87–5.12)	4.25 (3.62–5)	5.25 (4.25–6.25)	0.188
Left	3.25 (2–5.5)	2.5 (2–4.12)	5.5 (4–5.87)	0.114
CHFS (IQR)	39 (10.99–49.25)	17.08 (4.41–54.09)	44.5 (38–48.75)	0.551
DASH % (IQR)	59 (23–68)	44 (17–61)	66 (56–68)	0.220
HAQ (IQR)	1.37 (0.91–1.91)	1.37 (0.62–1.94)	1.56 (1.19–1.84)	0.663
SF-36 (IQR)				
Physical functioning	37.5 (30–61.25)	50 (30–76.25)	35 (31.25–38.75)	0.446
Limitations due to physical health	0 (0–56.25)	12.5 (0–62.5)	0 (0–37.5)	0.549
Limitations due to emotional problems	66.7 (0–100)	66.7 (8.33–91.67)	33.35 (0–91.67)	0.776
Energy/fatigue	42.5 (35–51.25)	45 (40–55)	37.5 (16.25–47.5)	0.156
Emotional well-being	56 (43–77)	70 (53–80)	42 (34–53)	0.073
Social functioning	50 (37.5–53.12)	50 (50–71.87)	37.5 (25–50)	**0.047**
Pain	45 (31.87–58.12)	45 (45–75)	37.5 (25–50)	0.270
General health	20 (15–36.25)	27.5 (16.25–43.75)	17.5 (7.5–27.5)	0.140

CHFS, Cochin Hand Function Scale; DASH, Disabilities of the Arm, Shoulder and Hand; FTP, finger-to-palm; HAMIS, Hand Mobility in Scleroderma; HAQ, health assessment questionnaire; IQR, interquartile range; SF-36, Short Form-36. Statistical significance is highlighted in bold.

**Table 3:** Changes in outcome measures from baseline (T0) to follow-up (T1) between the two groups.

	Intervention Group (*N* = 10)			Control Group (*N* = 6)		
	T0	T1	*p*	T0	T1	*p*
HAMIS (IQR)						
Right	12.5 (9.25–15)	10 (8.25–11)	**0.033**	17 (12–22)	17.5 (12.25–22.75)	0.893
Left	10.5 (7.25–13)	10.5 (7.5–13.5)	0.866	17.5 (12.5–21.75)	15.5 (10.5–20.5)	0.395
FTP distance, cm (IQR)						
Right	4.25 (3.62–5)	4 (3.12–4.5)	0.279	5.25 (4.25–6.25)	5.25 (4.62–5.5)	>0.99
Left	2.5 (2–4.12)	3 (2–4.47)	0.855	5.5 (4–5.87)	5.75 (4.75–6)	0.371
CHFS (IQR)	17.08 (4.41–54.09)	18 (10.75–48.25)	0.866	44.5 (38–48.75)	46 (43.5–57.5)	0.115
DASH % (IQR)	44 (17–61)	43 (21–58)	0.922	66 (56–68)	63 (58–83)	0.590
HAQ (IQR)	1.37 (0.62–1.94)	1.56 (0.72–2.03)	0.766	1.56 (1.19–1.84)	1.81 (1.53–2.47)	0.098
SF-36 (IQR)						
Physical functioning	50 (30–76.25)	62.5 (38.75–86.25)	0.114	35 (31.25–38.75)	37.5 (22.5–45)	0.890
Phys. H. Limit.	12.5 (0–62.5)	25 (25–62.5)	0.149	0 (0–37.5)	25 (6.25–43.75)	0.850
Em. Prob. Limit.	66.67 (8.33–91.67)	66.67 (33.33–100)	0.120	33.35 (0–91.67)	0 (0–75)	>0.99
Energy/fatigue	45 (40–55)	67.5 (55–80)	**0.008**	37.5 (16.25–47.5)	37.5 (12.5–43.75)	0.371
Emotional well-being	70 (53–80)	86 (81–88)	**0.009**	42 (34–53)	52 (40–61)	0.785
Social functioning	50 (50–71.87)	68.75 (40.62–84.37)	0.586	37.5 (25–50)	43.75 (9.37–50)	>0.99
Pain	45 (45–75)	60 (48.75–76.85)	**0.020**	37.5 (25–50)	38.75 (25–52.5)	>0.99
General health	27.5 (16.25–43.75)	32.5 (26.25–48.75)	**0.050**	17.5 (7.5–27.5)	10 (2.5–52.5)	0.181

CHFS, Cochin Hand Function Scale; DASH, Disabilities of the Arm, Shoulder and Hand; Em. Prob. Limit., Role limitation due to emotional problems; FTP, finger-to-palm; HAMIS, Hand Mobility in Scleroderma; HAQ, health assessment questionnaire; IQR, interquartile range; Phys. H. Limit., Role limitation due to physical health; SF-36, Short Form-36. Statistical significance is highlighted in bold.

**Table 4:** Between-group comparison of changes in outcome measures from baseline (T0) to follow-up (T1). Negative changes indicate improvement for HAMIS, FTP distance, CHFS, DASH and HAQ, while positive changes indicate improvement for SF-36 domains.

	Intervention Group (Mean Change, 95% CI)	Control Group (Mean Change, 95% CI)	*p*	Effect Size (Cohen’s d)	95% CI
HAMIS					
Right	−1.8 (−3.34; −0.26)	0.17 (−1.37; 1.71)	0.069	−1.02	−2.16; 0.20
Left	0.2 (−1.64; 2.04)	−0.83(−2.87; 1.20)	0.412	0.44	−0.63; 1.46
FTP distance, cm					
Right	−0.25 (−0.70; 0.20)	0 (−0.66; 0.66)	0.458	−0.39	−1.41; 0.66
Left	0.15 (−0.58; 0.88)	0.25 (−0.19; 0.69)	0.825	−0.12	−1.12; 0.90
CHFS	−0.43 (−4.62; 3.77)	6.17 (−0.64; 12.98)	0.055	−1.08	−2.25; 0.16
DASH	−0.5 (−8.47; 7.34)	3.6 (−6.57; 13.8)	0.457	−0.39	−1.42; 0.66
HAQ	0.06 (−0.20; 0.33)	0.27 (−0.02; 0.56)	0.255	−0.61	−1.66; 0.49
SF-36					
Physical functioning	8.00 (−2.82; 18.82)	−0.83 (−14.68; 13.01)	0.257	0.61	−0.49; 1.66
Limitations due to physical health	7.50 (−1.14; 16.14)	4.17 (−26.50; 34.84)	0.799	0.15	−0.86; 1.18
Limitations due to emotional problems	13.33 (−3.36; 30.00)	−11.11 (−39.67; 17.45)	0.077	0.99	−0.22; 2.12
Energy/fatigue	16.00 (7.95; 24.05)	−2.5 (−6.89; 1.89)	**0.002**	**1.97**	**0.38; 3.50**
Emotional well-being	17.60 (4.93; 30.27)	−2.67 (−12.51; 17.84)	0.104	0.90	−0.28; 2.01
Social functioning	3.75 (−8.93; 16.43)	0 (−18.55; 18.55)	0.688	0.21	−0.82; 1.22
Pain	9.75 (3.94; 15.55)	1.25 (−9.72; 12.22)	0.090	0.94	−0.25; 2.07
General health	5.50 (0.32; 10.68)	−5.83 (−14.24; 2.57)	**0.011**	**1.50**	**0.11; 2.83**

CHFS, Cochin Hand Function Scale; CI, confidence interval; DASH, Disabilities of the Arm, Shoulder and Hand; FTP, finger-to-palm; HAMIS, Hand Mobility in Scleroderma; HAQ, health assessment questionnaire; SF-36, Short Form-36. Mean changes were calculated as follow-up minus baseline (Δ = T1 − T0). Effect sizes follow the same direction as the corresponding outcome scales. Statistical significance is highlighted in bold.

## Data Availability

The raw data supporting the conclusions of this article will be made available by the authors on request.

## Supplementary Materials

The following supporting information can be downloaded at: https://www.mdpi.com/article/10.3390/jcm15187118/s1, Table S1. Sensitivity analysis of between-group differences in change scores using the Mann–Whitney U test. Negative changes indicate improvement for HAMIS, FTP distance, CHFS, DASH and HAQ, while positive changes indicate improvement for SF-36 domains.

## Abbreviations

The following abbreviations are used in this manuscript:

ATA	Anti-topoisomerase I antibody
CHFS	Cochin Hand Function Scale
CI	Confidence Interval
DASH	Disabilities of the Arm, Shoulder and Hand
FTP	Finger-to-palm
HAMIS	Hand Mobility in Scleroderma
HAQ	Health Assessment Questionnaire
IQR	Interquartile Range
mRSS	Modified Rodnan Skin Score
SCTC	Systemic Sclerosis Clinical Trials Consortium
SD	Standard Deviation
SF-36	Short Form-36
SMD	Standardized Mean Difference
SSc	Systemic Sclerosis
TFR	Tendon Friction Rubs
