# Supplementary material for: Effectiveness and Impact on Quality of Life of a Hand Rehabilitation Program in Longstanding Systemic Sclerosis: A Prospective, Non-Randomized, Controlled Pilot Study
Source: J Clin Med. 2026 Sep 14;15(18):7118. doi: 10.3390/jcm15187118 (PMC13607811; doi:10.3390/jcm15187118)
Supplement: Supplementary file 1 [file jcm-15-07118-s001.zip › jcm-4527215-supplementary.pdf]

**Supplementary Table S1.** Sensitivity analysis of between-group differences in change scores using the Mann–Whitney U test. Negative changes indicate improvement for HAMIS, FTP distance, CHFS, DASH and HAQ, while positive changes indicate improvement for SF-36 domains.

| | | Intervention group (mean change, 95% CI) | Control group (mean change, 95% CI) | p |
|---|---|---|---|---|
| HAMIS | | | | |
| | Right | -1.8 (-3.34; -0.26) | 0.17 (-1.37; 1.71) | 0.088 |
| | Left | 0.2 (-1.64; 2.04) | -0.83(-2.87; 1.20) | 0.323 |
| FTP distance, cm | | | | |
| | Right | -0.25 (-0.70; 0.20) | 0 (-0.66; 0.66) | 0.435 |
| | Left | 0.15 (-0.58; 0.88) | 0.25 (-0.19; 0.69) | 0.454 |
| CHFS | | -0.43 (-4.62; 3.77) | 6.17 (-0.64; 12.98) | 0.064 |
| DASH | | -0.5 (-8.47; 7.34) | 3.6 (-6.57; 13.8) | 0.562 |
| HAQ | | 0.06 (-0.20; 0.33) | 0.27 (-0.02; 0.56) | 0.209 |
| SF-36 | | | | |
| | Physical functioning | 8.00 (-2.82; 18.82) | -0.83 (-14.68; 13.01) | 0.270 |
| | Limitations due to physical health | 7.50 (-1.14; 16.14) | 4.17 (-26.50; 34.84) | 0.629 |
| | Limitations due to emotional problems | 13.33 (-3.36; 30.00) | -11.11 (-39.67; 17.45) | 0.157 |
| | Energy/fatigue | 16.00 (7.95; 24.05) | -2.5 (-6.89; 1.89) | **0.002** |
| | Emotional well-being | 17.60 (4.93; 30.27) | -2.67 (-12.51; 17.84) | 0.062 |
| | Social functioning | 3.75 (-8.93; 16.43) | 0 (-18.55; 18.55) | 0.776 |
| | Pain | 9.75 (3.94; 15.55) | 1.25 (-9.72; 12.22) | 0.086 |
| | General health | 5.50 (0.32; 10.68) | -5.83 (-14.24; 2.57) | **0.021** |

CHFS, Cochin Hand Function Scale; CI, confidence interval; DASH, Disabilities of the Arm, Shoulder and Hand; FTP, finger-to-palm; HAMIS, Hand Mobility in Scleroderma; HAQ, health assessment questionnaire; IQR, interquartile range; SF-36, Short Form-36. Statistical significance is highlighted in bold.

Mean changes were calculated as follow-up minus baseline ($\Delta = T1 - T0$). Mann–Whitney U tests were performed as sensitivity analyses because of the small sample size and departures from normality observed for some change-score distributions.
